# Pathogen-induced mitochondrial dysfunction: mechanistic insights, immune crosstalk, and therapeutic opportunities

**DOI:** 10.3389/fcimb.2025.1714998

**Published:** 2025-12-01

**Authors:** Yang Yang, Yuanyuan Zeng, Zeyi Liu, Jian-an Huang

**Affiliations:** 1 Department of Pulmonary and Critical Care Medicine, The First Affiliated Hospital of Soochow University, Suzhou, China; 2 Institute of Respiratory Diseases, Soochow University, Suzhou, China; 3 Suzhou Key Laboratory for Respiratory Diseases, Suzhou, China

**Keywords:** mitochondria, infection, mitochondrial dynamics, pathogens, host defense

## Abstract

Mitochondria have emerged as multifunctional organelles central to cellular metabolism, innate immunity, and cell fate determination. Increasing evidence demonstrates that pathogens-including viruses, bacteria, fungi, and parasites-target mitochondria to modulate host immune responses and metabolic reprogramming. Disruption of mitochondrial dynamics, excessive reactive oxygen species (ROS) generation, mitochondrial DNA (mtDNA) release, and altered mitophagy represent key hallmarks of pathogen-induced mitochondrial dysfunction. These processes not only compromise cellular bioenergetics but also influence immune signaling cascades, such as cGAS-STING and NLRP3 inflammasome pathways, thereby shaping infection outcomes. This review synthesizes the latest findings on how distinct pathogen classes orchestrate mitochondrial damage and explores their implications for infection biology and immune regulation. Furthermore, we highlight emerging mitochondria-targeted therapeutic strategies and future research directions aimed at mitigating infection-induced mitochondrial pathology.

## 1 Introduction

Mitochondria, traditionally recognized as cellular powerhouses, have evolved as dynamic signaling platforms regulating immunity, metabolism, and apoptosis. The importance of mitochondria extends beyond adenosine triphosphate (ATP) production to roles in calcium buffering, redox homeostasis, and coordination of innate immune signaling pathways (Stewart and Chinnery, 2015). Consequently, mitochondrial integrity is essential for cellular homeostasis and organismal survival. Over the past decade, mitochondria have emerged as crucial battlegrounds in the interplay between host defenses and invading pathogens. Pathogens ranging from viruses to multicellular parasites exhibit a remarkable ability to manipulate mitochondrial processes for their own advantage, either to dampen immune responses or exploit metabolic resources (Picard and Shirihai, 2022).

A growing body of research reveals that pathogen-induced mitochondrial damage is not a random event but rather a targeted, evolutionarily conserved strategy to optimize pathogen replication and persistence. The mechanisms underlying mitochondrial dysfunction include excessive generation of mitochondrial ROS (mtROS), loss of mitochondrial membrane potential, release of (mtDNA into the cytoplasm, and dysregulation of mitochondrial dynamics such as fission and fusion (Monzel et al., 2023). These processes not only compromise cellular bioenergetics but also trigger immune responses through sensors such as cyclic GMP-AMP synthase (cGAS) and stimulator of interferon genes (STING), or activate NOD-like receptor family pyrin domain-containing 3 (NLRP3) inflammasomes, leading to secretion of proinflammatory cytokines such as interleukin (IL)-1β (Mills et al., 2017).

The central role of mitochondria in immune regulation stems from the presence of mitochondrial antiviral signaling protein (MAVS), which serves as a critical adaptor for the RIG-I-like receptor (RLR) pathway, mediating type I interferon production upon viral RNA recognition (Tiku et al., 2020). Furthermore, mitochondrial ROS acts as a second messenger to activate inflammasomes and augment pathogen clearance. However, these same immune-activating features render mitochondria a prime target for pathogens seeking to evade host defenses. Viruses, for instance, encode proteases that cleave MAVS, whereas bacteria deploy toxins to permeabilize mitochondrial membranes, and fungi remodel host mitochondrial metabolism to withstand oxidative stress (Picard and Shirihai, 2022).

The strategies employed by pathogens to modulate mitochondrial function are strikingly diverse yet convergent in their ultimate goals: immune evasion and survival within the host. Viruses such as Severe acute respiratory syndrome coronavirus 2 (SARS-CoV-2), hepatitis C virus (HCV), and dengue virus manipulate mitochondrial dynamics and MAVS signaling to suppress antiviral responses (Zhou et al., 2023; Chen et al., 2025; Losarwar et al., 2025). Bacterial pathogens, including *Salmonella enterica* and *Helicobacter pylori*, utilize effectors and toxins to induce mitochondrial fragmentation and apoptosis (Wu et al., 2010; Zhang et al., 2024b). Fungal organisms, notably *Candida albicans* and *Aspergillus fumigatus*, reprogram mitochondrial respiration under oxidative stress conditions imposed by the host immune system (Fu et al., 2025; Ge et al., 2025). Protozoan parasites, including *Plasmodium falciparum* and *Trypanosoma cruzi*, exploit mitochondrial remodeling to acquire nutrients and modulate redox balance (Rodríguez-Hernández et al., 2020; Jain et al., 2024).

Mitochondrial dysfunction during infection is not merely a bystander effect; it significantly influences disease severity, chronic inflammation, and tissue pathology. In severe viral infections, uncontrolled mtDNA release can amplify systemic inflammation, contributing to cytokine storm syndromes, as observed in severe COVID-19 cases (Polli et al., 2025). Similarly, bacterial-induced mitochondrial permeability transition facilitates apoptosis and tissue damage, whereas impaired mitophagy during fungal or parasitic infections exacerbates host injury. Understanding these processes offers novel therapeutic opportunities: pharmacological interventions targeting mitochondrial ROS, promoting mitophagy, or modulating inflammasome activation represent promising avenues to mitigate infection-induced pathology (Gao et al., 2024; Jeon et al., 2025; Shen et al., 2025).

Current literature largely focuses on individual pathogen classes, with limited comparative analysis across viruses, bacteria, fungi, and parasites. Moreover, the therapeutic potential of mitochondria-targeted strategies remains underexplored in clinical settings. Addressing these gaps requires integrative approaches combining high-resolution imaging, omics technologies, and advanced infection models to delineate pathogen-specific and conserved mechanisms of mitochondrial manipulation.

This review provides a comprehensive synthesis of recent advances in understanding pathogen-induced mitochondrial damage. We systematically examine how distinct pathogen classes-viruses, bacteria, fungi, and parasites-target mitochondrial processes and discuss their immunological and pathological consequences. Furthermore, we highlight therapeutic interventions aimed at restoring mitochondrial homeostasis and outline future research directions in this rapidly evolving field.

## 2 Viral-induced mitochondrial damage mechanisms

Viruses have evolved sophisticated mechanisms to manipulate host cell organelles, with mitochondria being among the most strategically targeted structures. These organelles not only supply energy to sustain viral replication but also host critical signaling platforms for innate immunity, including MAVS and regulators of apoptotic pathways. Consequently, viruses exploit mitochondrial networks to subvert antiviral immunity, reprogram cellular metabolism, and ensure successful propagation. The following section delineates the major viral strategies leading to mitochondrial dysfunction, highlighting viral interference with innate immune signaling, mitochondrial dynamics, metabolic rewiring, and cell death pathways.

### 2.1 Viral suppression of MAVS-mediated antiviral signaling

MAVS, anchored on the outer mitochondrial membrane, is a key adaptor in the RLR signaling pathway that detects viral RNA. Upon activation by RIG-I or MDA5, MAVS initiates downstream signaling cascades leading to interferon regulatory factor 3 (IRF3) activation and production of type I interferons (IFNs), essential for antiviral defense (Liu and Gu, 2011; West et al., 2015). To evade immune detection, many RNA viruses deploy proteases to cleave MAVS or sequester it into inactive complexes.

HCV serves as a prime example: its NS3/4A protease cleaves MAVS at Cys508, releasing it from the outer mitochondrial membrane and disrupting RLR-mediated IFN signaling (Heim, 2013). Similarly, picornaviruses, including coxsackievirus B3 and enterovirus 71, employ 3C proteases to degrade MAVS, attenuating type I IFN responses (Mukherjee et al., 2011; Feng et al., 2014). Emerging evidence indicates that flaviviruses, such as dengue virus and Zika virus (ZIKV), also target MAVS by promoting its ubiquitination and proteasomal degradation (Zhang et al., 2018). These interactions demonstrate a convergent evolutionary strategy wherein viruses compromise mitochondrial innate immune hubs to suppress antiviral defenses.

SARS-CoV-2 encodes ORF9b, a small accessory protein that localizes to mitochondria, interacts with translocase of the outer membrane (TOM70), and impedes MAVS-dependent signaling (Gao et al., 2021; Wu et al., 2021). Structural analyses reveal that ORF9b binding to TOM70 disrupts the recruitment of heat shock protein 90 (Hsp90), impairing IRF3 phosphorylation and IFN induction (Zhou et al., 2024). This mechanism contributes to the blunted IFN responses observed in severe COVID-19 cases, correlating with hyperinflammation and poor clinical outcomes (Han et al., 2021).

### 2.2 Remodeling of mitochondrial dynamics to favor viral replication

Mitochondrial morphology, governed by dynamic fission and fusion events, profoundly influences cellular metabolism and apoptosis. Viruses exploit these processes to create a favorable intracellular environment. Fission, mediated by dynamin-related protein 1 (DRP1), facilitates mitochondrial fragmentation, often associated with apoptosis or mitophagy, while fusion, controlled by mitofusins (MFN1/2) and optic atrophy protein 1 (OPA1), promotes mitochondrial interconnectivity and bioenergetic stability (Zhang et al., 2018).

SARS-CoV-2 infection induces extensive mitochondrial fragmentation in epithelial and immune cells, attributed to upregulation and hyperactivation of DRP1 (Shin et al., 2024). Experimental inhibition of DRP1 using pharmacological agents such as Mdivi-1 mitigates mitochondrial fragmentation and reduces viral replication, underscoring the functional significance of mitochondrial dynamics during infection (Xu et al., 2025). Conversely, measles virus (MeV) and respiratory syncytial virus (RSV) promote mitochondrial elongation, delaying apoptosis and extending the survival of infected cells (Xia et al., 2014; Li et al., 2018). These findings highlight pathogen-specific modulation of mitochondrial dynamics, tailored to distinct replication strategies.

Interestingly, influenza A virus (IAV) infection has been shown to recruit PB1-F2, a proapoptotic viral protein, to the mitochondrial inner membrane, where it interacts with adenine nucleotide translocase 3 (ANT3) and disrupts mitochondrial integrity (Jaworska et al., 2014). PB1-F2 also interacts with TOM40, impairing protein import and contributing to mitochondrial stress (Yoshizumi et al., 2014). These alterations potentiate apoptosis, enhancing viral dissemination through tissue damage.

### 2.3 Viral induction of mitochondrial ROS to modulate host immunity and pathogenesis

Viral infections frequently induce oxidative stress by promoting excessive ROS generation, particularly from the mitochondrial electron transport chain (ETC). This oxidative burst serves dual roles: while moderate ROS levels activate antiviral signaling, excessive ROS production triggers mitochondrial permeability transition, mtDNA release, and cell death (Kayesh et al., 2025). Viruses exploit this delicate balance to modulate immune responses and pathogenesis.

HCV infection is strongly associated with mitochondrial ROS overproduction, driven by impaired electron flow in ETC complexes I and III and augmented calcium influx into mitochondria (Medvedev et al., 2016). ROS not only contributes to chronic liver inflammation but also potentiates carcinogenic processes by promoting lipid peroxidation and genomic instability (Uchida et al., 2020). SARS-CoV-2 infection similarly elevates mtROS, correlating with severe COVID-19 pathology characterized by cytokine storm and multi-organ failure (Nazerian et al., 2022). Mitochondria-targeted antioxidants, such as MitoQ, attenuate SARS-CoV-2-induced inflammatory cytokine production, suggesting therapeutic potential (Guarnieri et al., 2024).

### 2.4 Viral-induced release of mitochondrial DAMPs and innate immune activation

Mitochondrial damage during viral infection often leads to the release of mitochondrial damage-associated molecular patterns (DAMPs), such as mtDNA and cytochrome c, into the cytoplasm or extracellular milieu. mtDNA, enriched in unmethylated CpG motifs, activates pattern recognition receptors (PRRs) including cGAS and TLR9, amplifying inflammatory responses (Newman and Shadel, 2023). For example, IAV infection triggers mtDNA release via mitochondrial permeability transition pore (mPTP) opening, activating cGAS-STING signaling and promoting type I IFN responses (Moriyama et al., 2019). However, viruses can exploit this process: ZIKV induces mtDNA leakage while simultaneously inhibiting STING activation, skewing the immune response (Ding et al., 2018b).

Cytochrome c release, a hallmark of mitochondrial outer membrane permeabilization, activates the intrinsic apoptotic pathway via caspase-9 activation. Several viruses, including IAV and HIV-1, utilize mitochondrial apoptosis to facilitate viral spread, although some delay apoptosis early in infection to optimize replication (Galluzzi et al., 2008).

### 2.5 Viral-induced mtDNA release and cross-talk with innate immune signaling pathways

Once released into the cytosol, mitochondrial DNA (mtDNA) functions as a potent DAMP that activates multiple innate immune pathways. The cGAS-STING pathway senses cytosolic mtDNA and induces type I interferon (IFN-I) responses that restrict viral replication (West et al., 2015). In parallel, endosomal TLR9 recognizes mtDNA via CpG motifs, triggering MyD88-dependent NF-κB activation and the production of proinflammatory cytokines such as IL-6 and TNF-α (Costa et al., 2022).

Several viruses differentially modulate these responses: influenza virus enhances mtDNA release and TLR9 engagement, while herpes simplex virus type 1 inhibits TLR9 trafficking to evade detection. SARS-CoV-2 infection induces both cGAS-STING and TLR9 activation, contributing to the excessive cytokine production observed in severe COVID-19 (Nazerian et al., 2022). Together, these findings highlight that viral-induced mitochondrial stress engages a network of innate immune sensors-including cGAS and TLR9, interplay determines whether antiviral defense or pathological inflammation predominates.

### 2.6 Clinical implications and mitochondria-targeted therapeutic strategies in viral infections

Mitochondrial dysfunction during viral infections profoundly influences host immune balance, inflammation, and tissue integrity. Excessive mtROS production and mitochondrial fragmentation contribute to epithelial injury and cytokine amplification in influenza and COVID-19, promoting acute respiratory distress and multi-organ inflammation (Yeung-Luk et al., 2023; Yuan et al., 2023). Elevated circulating mtDNA levels correlate with disease severity and may serve as prognostic biomarkers in severe viral infections (Ali et al., 2020).

Chronic infections further exemplify the pathological role of mitochondrial disruption. In HBV and HCV, persistent mitochondrial stress drives hepatocyte apoptosis, fibrosis, and hepatocarcinogenesis (Kim et al., 2014; Li and Ou, 2023), whereas long-term antiretroviral therapy in HIV patients is associated with mitochondrial toxicity and metabolic syndrome (Pérez-Matute et al., 2013). Mitochondrial injury also contributes to cardiomyocyte loss and heart failure in viral myocarditis (Mohamud et al., 2023).

Therapeutically, restoring mitochondrial homeostasis represents a promising approach. Mitochondria-targeted antioxidants (MitoQ, SkQ1) and DRP1 inhibitors have shown potential in mitigating oxidative damage and improving outcomes in SARS-CoV-2 models (Shang et al., 2021; Guarnieri et al., 2024) Furthermore, modulation of mitophagy and metabolic signaling pathways is being explored to enhance antiviral immunity while limiting hyperinflammation (Song et al., 2025). Collectively, these findings highlight mitochondria as both biomarkers and actionable therapeutic targets to alleviate viral infection–associated pathology and improve clinical prognosis.

## 3 Bacterial-induced mitochondrial damage mechanisms

Mitochondria share an evolutionary lineage with α-proteobacteria, rendering them particularly susceptible to bacterial effectors and toxins. Bacterial pathogens exploit this evolutionary resemblance to target mitochondrial pathways, thereby subverting host cell metabolism, immune signaling, and apoptosis. Unlike viruses, which primarily manipulate host-encoded mitochondrial regulators, bacteria often deploy secreted toxins or effector proteins via specialized secretion systems to induce mitochondrial dysfunction. The resulting consequences-oxidative stress, permeability transition, mtDNA release, and apoptosis-profoundly impact disease pathogenesis. This section dissects the major mechanisms by which bacteria inflict mitochondrial damage, categorized into direct toxin effects, interference with mitochondrial dynamics, and modulation of innate immune signaling.

### 3.1 Bacterial toxins targeting mitochondrial structure and function

Several bacterial toxins exhibit direct mitochondrial tropism, targeting inner membrane components or ETC complexes to induce mitochondrial permeability transition and depolarization. Clostridium difficile toxins A and B, for example, disrupt mitochondrial integrity by inducing the opening of the mPTP, leading to mitochondrial swelling and cytochrome c release (Matarrese et al., 2007). *Helicobacter pylori*, a gastric pathogen implicated in peptic ulcers and gastric cancer, secretes the vacuolating cytotoxin (VacA), which localizes to the mitochondrial inner membrane and forms anion-selective channels. VacA insertion facilitates mitochondrial depolarization, swelling, and the release of pro-apoptotic factors such as apoptosis-inducing factor (AIF) and cytochrome c (Chatre et al., 2017).

*Listeria monocytogenes*, an intracellular pathogen, secretes listeriolysin O (LLO), a cholesterol-dependent cytolysin that destabilizes host membranes, including mitochondrial membranes (Carvalho et al., 2020). Sublytic concentrations of LLO promote mitochondrial fragmentation and mtROS accumulation, potentiating inflammasome activation (Li et al., 2021). Similarly, *Staphylococcus aureus* α-toxin and phenol-soluble modulins perturb mitochondrial function, amplifying tissue destruction during severe infections such as pneumonia and sepsis (Cheung et al., 2021).

### 3.2 Bacterial manipulation of mitochondrial dynamics

Mitochondrial fission and fusion processes, orchestrated by DRP1, MFN1/2, and OPA1, represent key checkpoints in cellular homeostasis. Several bacteria exploit these dynamics to manipulate host cell survival and immunity. *Legionella pneumophila*, the causative agent of Legionnaires’ disease, deploys a Type IV secretion system (T4SS) to translocate over 300 effectors into host cells. Among these, MitF and LncP have been shown to alter mitochondrial morphology by promoting fragmentation, thereby reducing host cell bioenergetic capacity and modulating apoptotic responses (Escoll et al., 2017).

*Salmonella enterica* serovar Typhimurium infection induces DRP1-mediated mitochondrial fragmentation through activation of host kinases that phosphorylate DRP1 at Ser616 (Fang et al., 2023). This fragmentation facilitates bacterial replication by impairing oxidative phosphorylation (OXPHOS) and redirecting cellular metabolism toward glycolysis-a metabolic state favorable for intracellular survival (Eisenreich et al., 2020). Pharmacological inhibition of DRP1 using Mdivi-1 attenuates *Salmonella* replication *in vitro*, highlighting the therapeutic potential of targeting mitochondrial dynamics (Liu et al., 2024).

Conversely, *Mycobacterium tuberculosis* (*M.tb*) infection stabilizes mitochondrial networks by inhibiting DRP1 activation, thus preventing premature host cell apoptosis and ensuring a replicative niche within macrophages (Xu et al., 2014). These contrasting strategies underscore pathogen-specific modulation of mitochondrial dynamics to optimize host-pathogen interactions.

### 3.3 Bacterial effectors modulating mitochondrial immune signaling pathways

Mitochondria are central hubs of innate immune signaling, particularly in the activation of MAVS-dependent antiviral responses and inflammasomes. Although MAVS primarily functions in antiviral defense, recent studies suggest cross-talk with antibacterial responses through mitochondrial ROS and NLRP3 inflammasome activation (Garaude et al., 2016). Several bacteria exploit this axis to dampen or hyperactivate immune responses.

*Listeria monocytogenes* and *Salmonella Typhimurium* manipulate NLRP3 inflammasome activity through mitochondrial perturbations. LLO-mediated mitochondrial damage promotes mtROS accumulation, a potent NLRP3 activator, leading to IL-1β secretion and pyroptosis (Diamond et al., 2017). While inflammasome activation contributes to pathogen clearance, excessive activation results in tissue damage and systemic inflammation, as seen in severe listeriosis (Eitel et al., 2010).

*Brucella abortus* deploys effector proteins such as BtpA, which interfere with mitochondrial metabolism and immune signaling, thereby suppressing proinflammatory cytokine production (Qin et al., 2024). *Legionella* effectors, including SidF, inhibit host apoptosis by antagonizing pro-apoptotic Bcl-2 family proteins, allowing prolonged intracellular survival (Banga et al., 2007).

### 3.4 Mitochondrial ROS and metabolic reprogramming in bacterial infection

Bacterial infections frequently induce mtROS as a byproduct of disrupted ETC function or calcium overload. While mtROS generation serves as a signaling cue for immune activation, excessive accumulation leads to oxidative stress, mtDNA damage, and mitochondrial permeability transition. *Helicobacter pylori* infection exemplifies this dual role: VacA-mediated mitochondrial dysfunction elevates ROS levels, activating NF-κB signaling and promoting chronic gastritis, while sustained oxidative stress predisposes to gastric carcinogenesis (Kim et al., 2018).

*Mycobacterium tuberculosis* infection profoundly reprograms host cell metabolism, promoting a shift from oxidative phosphorylation toward glycolysis, partly through inhibition of mitochondrial ETC activity (Kyung Kim and Jo, 2024). This metabolic rewiring favors bacterial persistence but also leads to bioenergetic failure and excessive ROS accumulation in infected macrophages (Spooner and Yilmaz, 2011). The role of mtROS in host-pathogen interactions extends to *Pseudomonas aeruginosa*, where quorum-sensing molecules induce mitochondrial depolarization and oxidative stress, contributing to tissue injury in chronic lung infections (Cutri et al., 2023).

### 3.5 Bacteria-induced mtDNA release and activation of innate immune

Mitochondrial damage induced by bacterial effectors often results in mtDNA leakage into the cytosol or extracellular environment. Cytosolic mtDNA serves as a potent activator of the cGAS-STING pathway, leading to type I IFN production. During *Listeria monocytogenes* infection, LLO promotes mitochondrial membrane permeabilization, facilitating mtDNA release and subsequent activation of the cGAS-STING pathway (Chauhan et al., 2024). This signaling contributes to early antibacterial defense by enhancing IFN-β production; however, sustained mitochondrial injury can aggravate inflammation, underscoring the context-dependent dual role of mitochondrial alterations in host-pathogen interactions.

Similarly, *Salmonella enterica* serovar Typhimurium infection induces mitochondrial stress and mtDNA release in murine macrophages, thereby activating the cGAS-STING-dependent type I IFN response (Xu et al., 2022). This pathway enhances early innate immune recognition but can also promote excessive inflammatory signaling if not tightly regulated. Interestingly, some bacterial pathogens have evolved mechanisms to counteract mtDNA-mediated immune activation. For example, *Legionella pneumophila* secretes the effector SdhA, which maintains mitochondrial integrity and limits mtDNA leakage, helping the bacterium to avoid immune hyperactivation (Creasey and Isberg, 2012).

Beyond the cGAS-STING and NLRP3 inflammasome pathways, mtDNA also serves as a ligand for endosomal TLR9, which recognizes unmethylated CpG motifs in bacterial and mitochondrial DNA (Bauer et al., 2001; Tripathi et al., 2023). Activation of the TLR9-MyD88 signaling axis leads to NF-κB activation and the production of proinflammatory cytokines such as IL-6 and TNF-α, further amplifying antibacterial responses. However, excessive or dysregulated TLR9 activation has been implicated in sepsis-related organ injury (Cheng et al., 2020), Thus, mitochondrial manipulation by pathogens may not only influence the cGAS-STING and NLRP3 pathways but also extend to TLR9-MyD88 signaling, further diversifying immune outcomes.

Importantly, bacteria-induced mitochondrial dysfunction can trigger multiple forms of regulated cell death beyond apoptosis. Excessive mitochondrial ROS generation and mtDNA release can activate pyroptosis through NLRP3 inflammasome assembly and gasdermin D pore formation (Paik et al., 2025). Disruption of mitochondrial complex I and metabolic stress can also promote necroptosis, mediated by RIPK3-dependent pathways observed in *Salmonella* and *Listeria* infections (Sai et al., 2019; Dong et al., 2022). Moreover, bacterial toxins that alter iron metabolism or enhance lipid peroxidation may induce ferroptosis, a mitochondria-associated, iron-dependent cell death modality (Chen et al., 2021). These processes not only eliminate infected cells but can also amplify inflammation, influencing bacterial persistence and host pathology.

Collectively, these findings demonstrate that bacterial-induced mitochondrial dysfunction serves as a central signaling hub that coordinates innate immune activation and cell death programs. The balance between protective and detrimental mitochondrial responses ultimately determines whether infection resolves or progresses toward immunopathology.

### 3.6 Clinical implications and therapeutic opportunities in bacterial infections

Bacterial-induced mitochondrial damage has profound implications that extend beyond localized cellular dysfunction to systemic disease outcomes. Excessive mtROS production and subsequent activation of inflammasomes play a critical role in the pathophysiology of sepsis, driving uncontrolled inflammation and multi-organ failure. Chronic infections, including those caused by *Helicobacter pylori* and *Mycobacterium tuberculosis*, exploit mitochondrial manipulation to facilitate long-term persistence within the host, thereby evading immune clearance (Odoom et al., 2025). These observations collectively position mitochondria as a central node in host-pathogen interactions and highlight their potential as strategic therapeutic targets in bacterial infections.

Therapeutic strategies designed to counteract pathogen-induced mitochondrial dysfunction focus on restoring mitochondrial integrity and immune balance. Mitochondria-targeted antioxidants, such as MitoQ and SkQ1, can alleviate oxidative stress and reduce tissue damage (Nath et al., 2025). DRP1 inhibitors have been proposed to restrict pathological mitochondrial fragmentation during *Salmonella* infection, thereby preserving mitochondrial function (Liu et al., 2024). For *Helicobacter pylori*-associated gastric pathology, VacA channel blockers offer a promising approach to prevent toxin-mediated mitochondrial disruption (Chen et al., 2016).

In addition, the polyphenol epigallocatechin gallate (EGCG) has been shown to suppress cGAS activation, thereby mitigating Carbapenem-resistant *Acinetobacter baumannii*-induced inflammation and mitochondrial dysfunction, highlighting its potential as a host-directed immunomodulator (Yang et al., 2024). Likewise, the NLRP3-specific inhibitor MCC950 effectively reduces IL-1β release induced by bacterial lipopolysaccharides, providing an additional means to limit excessive inflammasome activation and cytokine storm (Coll et al., 2015). Collectively, these findings underscore the therapeutic promise of host-directed therapies (HDTs) that integrate conventional antibiotics with mitochondrial protectants and immune modulators, offering innovative approaches to enhance treatment efficacy and combat drug-resistant bacterial infections.

## 4 Fungal-induced mitochondrial damage mechanisms

Fungal pathogens, ranging from opportunistic yeasts such as *Candida albicans* to filamentous species like *Aspergillus fumigatus*, impose substantial burdens on immunocompromised hosts. Although mitochondria have long been recognized as key regulators of host cell homeostasis, their role in antifungal immunity and fungal pathogenesis is gaining increasing attention. Unlike viruses and bacteria, fungi do not typically inject effectors into host cells through specialized secretion systems; however, they secrete an array of toxins, secondary metabolites, and enzymes that profoundly influence mitochondrial function. Moreover, mitochondrial dysfunction in immune cells can alter antifungal responses, facilitating persistence and dissemination. This section dissects major mitochondrial alterations during fungal infections, focusing on oxidative stress, metabolic reprogramming, mitochondrial dynamics, and innate immune signaling.

### 4.1 Interaction between fungal virulence factors and host mitochondria

Fungal virulence factors directly target host mitochondria, profoundly disrupting bioenergetic and immune functions. Among these, *Aspergillus fumigatus*–derived gliotoxin is a well-characterized mitochondrial toxin that inhibits electron transport chain (ETC) complexes, induces excessive ROS generation, and triggers apoptosis in epithelial and immune cells (Warris and Ballou, 2019). Similarly, candidalysin-a pore-forming peptide secreted by hyphal *Candida albicans*-integrates into host and mitochondrial membranes, leading to depolarization, cytochrome c release, and activation of apoptotic pathways (Spaggiari et al., 2024).

Additionally, *Candida albicans* secretes aspartyl proteinases (SAPs) that degrade mitochondrial structural and metabolic proteins, further impairing mitochondrial integrity and energy production (Chakraborty et al., 2023). *Cryptococcus neoformans* utilizes a polysaccharide capsule to suppress mitochondrial metabolism in macrophages, decreasing mtROS production and blunting fungicidal activity (Coelho et al., 2015). these strategies underscore mitochondria as key cellular targets for fungal immune evasion and persistence, laying the foundation for subsequent mitochondrial dysfunction, oxidative stress, and dysregulated immune signaling during fungal infection.

### 4.2 Fungal-mediated disruption of mitochondrial bioenergetics and ETC function

Mitochondrial OXPHOS serves as a key energy source for immune cell function, particularly for macrophages and dendritic cells engaged in antifungal defense. Fungal infections often impair OXPHOS by disrupting ETC components. In *Candida* infections, host mitochondria exhibit decreased complex I and complex III activity, correlating with reduced ATP levels and impaired phagocytic killing capacity (Li and Calderone, 2017). This mitochondrial dysfunction shifts cellular metabolism toward aerobic glycolysis (Warburg-like effect), promoting a pro-inflammatory phenotype that, while beneficial for fungal clearance in acute infection, can exacerbate immunopathology during chronic infection (Braunstein et al., 2025).

Moreover, gliotoxin from *Aspergillus fumigatus* and candidalysin from *Candida albicans* synergistically impair mitochondrial ATP production, weakening epithelial barrier integrity and facilitating fungal invasion (Brown et al., 2021). Such disruptions underscore mitochondria as central nodes in host-pathogen metabolic interactions.

### 4.3 Fungal-induced mitochondrial ROS generation and oxidative stress

One of the earliest mitochondrial responses to fungal infection is the excessive generation of mtROS. During *Candida albicans* infection, phagocytic cells such as neutrophils and macrophages mount oxidative bursts to kill invading fungi. However, this defense mechanism can inadvertently amplify mtROS production within host mitochondria, leading to oxidative stress and mitochondrial damage (Sobén et al., 2025). Excessive mtROS can lead to mitochondrial membrane depolarization, increase the mPTP, and facilitate the release of cytochrome c along with other pro-apoptotic factors (Spaggiari et al., 2024).

Conversely, fungi themselves exploit ROS as both signaling molecules and virulence factors. For instance, *Aspergillus fumigatus* produces gliotoxin, a secondary metabolite with strong immunosuppressive and mitochondrial-disruptive properties. Gliotoxin directly targets mitochondrial respiratory complexes, impairing ETC function and inducing ROS overproduction (Warris and Ballou, 2019). Elevated ROS levels compromise mitochondrial integrity and activate cell death pathways, contributing to pulmonary tissue damage observed in invasive aspergillosis (Xu et al., 2020). In addition, gliotoxin inhibits NF-κB activation in immune cells, blunting antifungal immunity (Brown et al., 2021).

### 4.4 Fungal modulation of mitochondrial dynamics and mitophagy in host defense

Mitochondrial morphology, governed by the balance between fission and fusion, adapts dynamically to cellular stress. During fungal infections, mitochondrial fragmentation (fission) is frequently observed, often preceding apoptotic or pyroptotic cell death (Pardo et al., 2006). *Candida albicans* infection activates DRP1, promoting mitochondrial fission and loss of mitochondrial membrane potential in epithelial cells (Mao et al., 2021). Excessive fission can trigger apoptosis and epithelial barrier disruption, aiding fungal dissemination.

In contrast, fusion events mediated by MFN1/2 can maintain mitochondrial integrity under moderate stress. Experimental inhibition of DRP1 using the small-molecule inhibitor Mdivi-1 attenuates *Candida*-induced epithelial cell apoptosis, suggesting a potential therapeutic strategy (Koch and Traven, 2019).

Mitophagy-the selective autophagic degradation of damaged mitochondria-emerges as a key quality-control mechanism during fungal infections. In macrophages exposed to conidia, mitophagy is activated to remove ROS-damaged mitochondria and prevent excessive inflammasome activation (Garrido-Bazán et al., 2020). However, certain fungal pathogens can exploit this process; for instance, *Cryptococcus neoformans* infection induces excessive mitophagy, blunting immune cell activation and cytokine production (Ding et al., 2018a). Thus, mitophagy represents a double-edged sword in antifungal immunity.

### 4.5 Fungal-induced mitochondrial signaling and innate immune activation

Mitochondria serve as signaling platforms for innate immune pathways such as MAVS-dependent antiviral signaling, the NLRP3 inflammasome, and cGAS-STING. Although MAVS is traditionally linked to antiviral immunity, mitochondrial dysfunction during fungal infections can indirectly influence these pathways. For example, mtROS and oxidized mtDNA released during Candida infection serve as potent activators of the NLRP3 inflammasome, driving IL-1β and IL-18 secretion (Westman et al., 2020). NLRP3 activation is critical for antifungal defense; mice deficient in NLRP3 exhibit increased susceptibility to systemic candidiasis (Loh and Lam, 2023). However, uncontrolled inflammasome activation can exacerbate tissue damage, contributing to immune pathology in disseminated fungal infections (Skeldon and Saleh, 2011).

Recent studies indicate that *Aspergillus fumigatus* infection can induce mtDNA leakage into the cytosol, activating cGAS-STING signaling and type I interferon production (Peng et al., 2023). Although type I IFNs have ambiguous roles in fungal infections, their induction suggests mitochondria as key immunomodulatory hubs during fungal challenge.

### 4.6 Clinical implications and therapeutic perspectives in fungal infections

Mitochondrial dysfunction during fungal infections contributes to both immune dysregulation and tissue damage, presenting opportunities for therapeutic intervention. Excessive mtROS and disrupted mitochondrial dynamics amplify inflammation and impair host defense. Mitochondria-targeted antioxidants such as MitoQ and SkQ1 have shown efficacy in experimental models of candidiasis and aspergillosis, alleviating oxidative stress and preserving mitochondrial integrity (Mukherjee et al., 2024). Similarly, inhibitors of DRP1-mediated fission, such as Mdivi-1, show promise in reducing epithelial damage during *Candida* infection (Koch et al., 2018).

Beyond antioxidant approaches, restoring mitochondrial quality control has emerged as a complementary therapeutic avenue. Modulators of mitophagy, including PINK1/Parkin activators, may enhance the clearance of damaged mitochondria and promote antifungal immunity, although their therapeutic potential requires further validation (Chen et al., 2019). In parallel, combination therapies targeting both fungal growth and mitochondrial homeostasis represent a promising strategy. For example, co-administration of antifungal agents with mitochondrial protectants can improve outcomes in immunocompromised patients while reducing inflammatory complications (Qin et al., 2023). Such integrated interventions may offer synergistic benefits by addressing both pathogen burden and host mitochondrial resilience.

Recent studies further highlight the immunomodulatory potential of mitochondrial signaling in antifungal defense. Mitochondrial-derived reactive oxygen species and oxidized mtDNA are potent activators of the NLRP3 inflammasome, driving maturation of IL-1β and IL-18, which in turn enhance Th1 and Th17 responses essential for protection against *Candida albicans* and *Aspergillus fumigatus* (Hise et al., 2009; Gross et al., 2011; Patel et al., 2018). Controlled activation of these pathways could thus be harnessed in vaccine adjuvant design. For instance, β-glucan-based formulations have been shown to prime mitochondrial metabolism and NLRP3 activation in macrophages, improving vaccine efficacy while maintaining immune balance (Qin et al., 2023). Collectively, these insights suggest that fine-tuning mitochondrial signaling may offer a dual advantage‐enhancing antifungal immunity and minimizing immunopathology.

## 5 Parasite-induced mitochondrial damage mechanisms

Parasitic infections, caused by protozoa such as *Plasmodium* spp., *Leishmania* spp., *Trypanosoma* spp., and *Toxoplasma gondii*, represent a global health challenge, particularly in tropical and subtropical regions. These organisms exhibit complex life cycles and highly specialized strategies for survival within host cells. Unlike many bacteria and fungi, protozoan parasites can reside intracellularly, enabling direct interactions with host organelles, including mitochondria. Increasing evidence suggests that parasites manipulate mitochondrial function to secure nutrients, modulate immune responses, and evade host defenses. This section explores how parasites alter mitochondrial physiology, focusing on bioenergetic reprogramming, oxidative stress, mitochondrial dynamics, and immune signaling.

### 5.1 Parasite virulence factors targeting host mitochondria

Several parasitic effector molecules directly manipulate host mitochondrial function. *Toxoplasma gondii* secretes dense granule proteins (GRAs) such as GRA7 and GRA14, which mediate mitochondrial recruitment and alter organelle morphology (Sasai and Yamamoto, 2022). GRA15, another effector, modulates NF-κB signaling through mitochondrial interactions (Rosowski et al., 2011). *Plasmodium* exports proteins that affect host hepatocyte metabolism, including those influencing mitochondrial fatty acid oxidation (Ibitokou et al., 2018). Leishmania lipophosphoglycan (LPG) interacts with mitochondrial membranes, reducing mitochondrial membrane potential and impairing ROS production (Rodrigues et al., 2025). These strategies underscore mitochondria as central hubs targeted by parasite virulence mechanisms.

### 5.2 Parasite-induced reprogramming of host mitochondrial bioenergetics

Parasites have evolved mechanisms to subvert host mitochondrial metabolism to meet their energy and biosynthetic needs. *Plasmodium* falciparum, the causative agent of malaria, induces profound metabolic shifts in hepatocytes and erythrocytes during its intrahepatic stage. Although red blood cells lack mitochondria, hepatocytes exhibit altered OXPHOS and tricarboxylic acid (TCA) cycle activity upon parasite infection (Evers et al., 2021). Studies using metabolic flux analysis reveal that *Plasmodium* infection decreases host mitochondrial respiration while increasing glycolytic flux, mirroring the Warburg-like effect seen in cancer cells (Nair et al., 2023). This metabolic remodeling supports parasite replication by reallocating resources such as amino acids and lipids.

Similarly, *Toxoplasma gondii*, an obligate intracellular protozoan, forms a parasitophorous vacuole (PV) within host cells, which closely associates with host mitochondria in a process termed host mitochondrial association (HMA) (Barik and Andrews, 2024). HMA facilitates the parasite’s access to host-derived metabolites, including fatty acids and ATP, while also influencing mitochondrial signaling pathways. Genetic disruption of HMA in *Toxoplasma gondii* reduces parasite growth and virulence, underscoring the importance of mitochondrial hijacking for parasitic survival (Powell et al., 2024).

*Leishmania donovani*, the etiological agent of visceral leishmaniasis, infects macrophages and modulates their energy metabolism to favor a permissive niche. Infected macrophages display reduced mitochondrial membrane potential and decreased ATP levels, leading to impaired antimicrobial effector functions (de Oliveira et al., 2025). This bioenergetic compromise enhances parasite survival while dampening host defense.

### 5.3 Parasite-induced oxidative stress and mitochondrial dysfunction

Parasite-induced oxidative stress plays a dual role in infection dynamics. On one hand, host immune cells generate ROS as part of their antimicrobial arsenal; on the other hand, excessive ROS can damage host mitochondria, impair immune function, and promote parasite persistence. *Leishmania* spp. infection, for instance, triggers mitochondrial ROS production in macrophages, leading to mitochondrial depolarization and activation of apoptosis pathways (Ball et al., 2014). However, parasites have evolved antioxidant systems such as trypanothione reductase and superoxide dismutase to neutralize ROS, thereby reducing oxidative damage to themselves while allowing controlled mitochondrial stress in host cells (Basu Ball et al., 2011).

*Toxoplasma gondii* infection also induces oxidative stress by disrupting host mitochondrial ETC activity. Experimental models demonstrate that *Toxoplasma gondii*-infected cells exhibit increased mtROS levels, which activate autophagic pathways and promote nutrient mobilization beneficial to the parasite (Periyasamy-Thandavan et al., 2009).

*Plasmodium* infections, particularly during the blood stage, are associated with systemic oxidative stress, partly due to hemoglobin degradation and heme release. Although mature erythrocytes lack mitochondria, liver-stage parasites influence hepatocyte mitochondrial ROS dynamics. Elevated mtROS during this phase may contribute to hepatocyte apoptosis and liver pathology observed in severe malaria cases (Kaushansky et al., 2013).

### 5.4 Parasite-mediated modulation of mitochondrial dynamics and mitophagy

Mitochondrial dynamics, encompassing fission, fusion, and mitophagy, undergo significant alterations during parasitic infections. Studies in *Toxoplasma gondii*-infected cells reveal marked mitochondrial fragmentation mediated by DRP1 activation (Melatti et al., 2019). Excessive fission promotes mitochondrial depolarization and cytochrome c release, favoring apoptosis in certain cell types, while in others, it may facilitate parasite access to mitochondrial lipids. Pharmacological inhibition of DRP1 with Mdivi-1 reduces mitochondrial fragmentation and partially restores cellular homeostasis, although its impact on parasite survival remains variable (Smirnova et al., 2001).

Conversely, mitophagy emerges as a host defense mechanism to eliminate damaged mitochondria during parasitic infections. In macrophages infected with *Leishmania donovani*, activation of PINK1/Parkin-mediated mitophagy reduces mtROS accumulation and prevents uncontrolled inflammasome activation (Giri and Shaha, 2019). However, excessive mitophagy may deplete mitochondrial mass and impair immune cell energy supply, ultimately favoring parasite persistence. This dual role positions mitophagy as a potential therapeutic target for modulating host-parasite interactions.

### 5.5 Parasite-induced mitochondrial signaling and immune evasion

Mitochondria orchestrate several innate immune pathways critical for parasite control, including NLRP3 inflammasome activation and type I interferon responses. mtROS and oxidized mtDNA serve as potent triggers of the NLRP3 inflammasome, leading to caspase-1 activation and IL-1β secretion (Alonaizan, 2024). While inflammasome activation enhances parasite clearance, excessive activation can drive immunopathology. *Leishmania* spp. infection dampens NLRP3 activation by modulating mitochondrial ROS production, partly through upregulation of antioxidant enzymes such as superoxide dismutases (SOD1 and SOD2), catalase (CAT), and glutathione peroxidase (GPx). These enzymes act synergistically to scavenge superoxide and hydrogen peroxide, thereby limiting ROS-mediated NLRP3 activation and promoting parasite persistence (Harrington and Gurung, 2020).

*Toxoplasma gondii* also interferes with mitochondrial signaling to evade immune detection. By recruiting host mitochondria to its parasit ophorous vacuole, *Toxoplasma gondii* prevents the release of mitochondrial danger-associated molecular patterns (DAMPs) such as mtDNA, which would otherwise activate cytosolic DNA sensors like cGAS-STING (Jin et al., 2023). Moreover, parasites secrete effectors that inhibit MAVS signaling on mitochondria, thereby blunting interferon responses (Zhang et al., 2022).

### 5.6 Clinical implications and therapeutic opportunities in parasitic infections

Understanding parasite-induced mitochondrial dysfunction opens new therapeutic horizons. Targeting mitochondrial quality-control pathways, such as enhancing mitophagy in early infection while preventing excessive mitochondrial loss, could optimize immune function. Mitochondria-targeted antioxidants like MitoQ and SkQ1 show promise in reducing oxidative damage and restoring cellular homeostasis during parasitic infections (Long et al., 2012). Additionally, inhibition of excessive mitochondrial fission with the DRP1 inhibitor Mdivi-1 can alleviate mitochondrial fragmentation and partially restore cellular homeostasis, highlighting mitochondrial dynamics as a druggable target (Thakur et al., 2025).

Drug repurposing approaches have also explored the use of mitochondrial inhibitors to selectively target parasites. For instance, atovaquone, a known antimalarial, acts by inhibiting parasite mitochondrial cytochrome bc1 complex, demonstrating the feasibility of mitochondria-focused antiparasitic strategies (Sheokand et al., 2024). Combining such agents with host-directed mitochondrial protectants could synergistically enhance therapeutic efficacy.

Emerging research suggests that mitochondrial signaling pathways could be leveraged as adjuvants in antiparasitic vaccines. For example, controlled activation of NLRP3 inflammasome or cGAS-STING pathways via mitochondrial DAMP mimetics might boost protective immunity without causing excessive inflammation (Sun and Cheng, 2020; Alonaizan, 2024).

Collectively, these findings underscore mitochondria as central therapeutic targets in parasite-host interactions. By integrating antioxidants, mitophagy regulators, and immunomodulators, future mitochondria-focused strategies hold promise for limiting infection-induced tissue damage, enhancing antiparasitic immunity, and overcoming the challenge of drug resistance in parasitic diseases.

## 6 Integrative discussion and future perspectives

The intricate interplay between mitochondrial dysfunction and pathogen infection highlights mitochondria as a central hub for host-pathogen interactions. Across diverse pathogens-viruses, bacteria, fungi, and parasites-common themes emerge, including disruption of mitochondrial bioenergetics, induction of oxidative stress, modulation of mitochondrial dynamics, and interference with immune signaling pathways. These perturbations not only facilitate pathogen survival and replication but also shape disease severity and host outcomes, a cross-pathogen comparison that is summarized in **Table 1**. This section synthesizes mechanistic insights, compares cross-pathogen strategies, proposes visual representations for key concepts, and explores therapeutic implications and future research directions.

**Table 1:** Summary of pathogen-induced mitochondrial alterations and their functional consequences.

Pathogen class	Representative pathogen/effector	Targeted mitochondrial process	Outcome/host effect	References
Viruses	Hepatitis C virus (NS3/4A), SARS-CoV-2 (ORF9b), Influenza A (PB1-F2)	MAVS cleavage or degradation; inhibition of mitophagy; mitochondrial depolarization; ROS overproduction	Impaired type I IFN signaling; cGAS-STING activation via mtDNA release; NLRP3 inflammasome activation; apoptosis	(Jaworska et al., 2014; Medvedev et al., 2016; Gao et al., 2021)
Bacteria	*Helicobacter pylori* (VacA), *Listeria monocytogenes* (LLO), *Salmonella enterica* (SipA)	Mitochondrial membrane permeabilization; mtDNA release; DRP1-mediated fission; inhibition of mitophagy	Activation of cGAS-STING, TLR9, and NLRP3 pathways; cytokine release (IL-1β, IFN-β); inflammation and tissue damage	(Chatre et al., 2017; Carvalho et al., 2020; Liu et al., 2024)
Fungal	*Candida albicans*, *Aspergillus fumigatus* (gliotoxin), *Cryptococcus neoformans*	Mitochondrial depolarization; electron transport chain inhibition; excessive ROS generation; mitophagy impairment	NLRP3 inflammasome activation; metabolic reprogramming; tissue necrosis and immune evasion	(Ding et al., 2018a; Brown et al., 2021; Sobén et al., 2025)
Parasites	*Toxoplasma gondii* (GPRs), *Plasmodium falciparum, Leishmania donovani* (LPG),	Disturbed mitochondrial membrane potential; ROS imbalance; altered fission/fusion; mtDNA release	Macrophage polarization shift; cytokine dysregulation; chronic inflammation; immune exhaustion	(Kaushansky et al., 2013; Ball et al., 2014; Jin et al., 2023)

### 6.1 Cross-pathogen mechanistic convergence and divergence in mitochondrial dysfunction

Although distinct in biology, viruses, bacteria, fungi, and parasites exploit overlapping mitochondrial vulnerabilities. Mitochondrial bioenergetics reprogramming is a unifying strategy. Viruses such as SARS-CoV-2 and influenza A shift host energy metabolism toward glycolysis while reducing OXPHOS, ensuring rapid biomass production favorable for replication (Shin et al., 2024). Similarly, intracellular bacteria like Mycobacterium *tuberculosis* and *Listeria monocytogenes* impair mitochondrial respiration to dampen immune activation (Spier et al., 2021). Fungi and protozoa also induce bioenergetic changes; for example, *Candida albicans* infection compromises OXPHOS in macrophages, whereas *Toxoplasma gondii* physically hijacks host mitochondria to scavenge metabolites (Lee et al., 2005; Zhang et al., 2022).

Oxidative stress represents another shared mechanism. Elevated mtROS during infection acts as both a signaling intermediate and a pathogenic driver. While moderate ROS boosts antimicrobial responses via NLRP3 inflammasome activation, uncontrolled mtROS exacerbates tissue injury, as observed in severe viral pneumonia, tuberculosis granulomas, and systemic candidiasis (Dan Dunn et al., 2015). Pathogens counterbalance this stress using antioxidant enzymes or by promoting mitophagy, highlighting the evolutionary arms race over mitochondrial homeostasis.

In contrast, pathogen-specific strategies reflect divergent evolutionary pressures. Viruses frequently manipulate MAVS signaling on mitochondria to evade type I interferon responses (Belgnaoui et al., 2011), whereas bacteria such as Salmonella exploit mitochondrial fission to induce apoptosis and escape immune clearance (Tiku et al., 2020). Fungal pathogens employ mitochondrial adaptation to hypoxic niches, while parasites engage in HMA to siphon energy resources (Pernas et al., 2014; Pradhan et al., 2018). These differences underscore the necessity for tailored interventions while maintaining awareness of shared vulnerabilities.

### 6.2 Immune-mitochondrial crosstalk in infection

Pathogen-induced mitochondrial damage and immune-mediated mitochondrial stress form an intricate feedback network within the infection microenvironment. While pathogens directly disrupt mitochondrial homeostasis through toxins, viral proteins, or metabolic hijacking, the ensuing immune activation further amplifies mitochondrial dysfunction. Proinflammatory cytokines such as TNF-α, IL-1β, and IFN-γ can impair mitochondrial respiration, increase ROS production, and trigger mitochondrial permeability transition, leading to secondary mitochondrial stress in immune and parenchymal cells (West and Shadel, 2017; Tiku et al., 2020). Activated macrophages and neutrophils generate large amounts of ROS and nitric oxide as antimicrobial defenses, but excessive production can exacerbate mitochondrial oxidative damage (Weinberg et al., 2015).

Moreover, mitochondrial DAMPs released from stressed cells-such as mtDNA and cardiolipin-serve as potent activators of pattern recognition receptors (e.g., TLR9, cGAS-STING, and NLRP3), perpetuating inflammation (Mills et al., 2017). Thus, infection-induced mitochondrial dysfunction and immune activation are reciprocally reinforcing processes that create a vicious cycle of oxidative stress, metabolic reprogramming, and immunopathology. Targeting this feedback loop represents a promising therapeutic direction to restore immune–metabolic balance during infection.

### 6.3 Tissue- and metabolic context-dependent mitochondrial responses to infection

Mitochondrial responses to infection are not uniform but vary according to tissue type, cell lineage, and the host’s metabolic state. Highly oxidative organs such as the heart, liver, and brain are particularly vulnerable to pathogen-induced mitochondrial dysfunction due to their dependence on oxidative phosphorylation. For example, *Mycobacterium tuberculosis* infection induces distinct mitochondrial transcriptional responses in alveolar macrophages compared with hepatocytes, reflecting differences in metabolic programming and immune function (Russell et al., 2019). Similarly, *Candida albicans* infection elicits more pronounced mitochondrial fragmentation in epithelial cells, consistent with cell type-specific thresholds for mitophagy activation (Blagojevic et al., 2021).

Host metabolic status further modulates these responses. Ageing, obesity, and diabetes are associated with basal mitochondrial dysfunction and chronic low-grade inflammation, which can exacerbate infection-induced mitochondrial stress (Picca et al., 2022). Age-related decline in mitophagy and antioxidant capacity may increase susceptibility to severe infections such as influenza and sepsis. Therefore, understanding tissue- and metabolism-dependent heterogeneity in mitochondrial signaling is crucial for translating mechanistic insights into clinical applications. Such knowledge could inform the design of precision therapies that restore mitochondrial homeostasis in a tissue-specific manner while minimizing systemic side effects.

### 6.4 Mitochondrial quality control: balancing protection and pathology during infection

Mitophagy and mitochondrial biogenesis represent critical quality control processes. During infection, selective removal of damaged mitochondria via PINK1/Parkin-dependent mitophagy limits mtROS accumulation and prevents excessive inflammasome activation (Cho et al., 2020). However, hyperactivation of mitophagy, as seen in chronic viral hepatitis or Leishmania infection, depletes mitochondrial mass, impairing ATP production and immune competence (Cho et al., 2020). Conversely, suppression of mitophagy leads to persistent oxidative stress and inflammatory damage, evident in severe COVID-19 and fungal sepsis (Xu et al., 2024). Thus, therapeutic modulation of mitophagy requires fine-tuning rather than blanket activation or inhibition.

### 6.5 Mitochondria as a central platform for immune signaling and pathogen recognition

**Figure 1:**
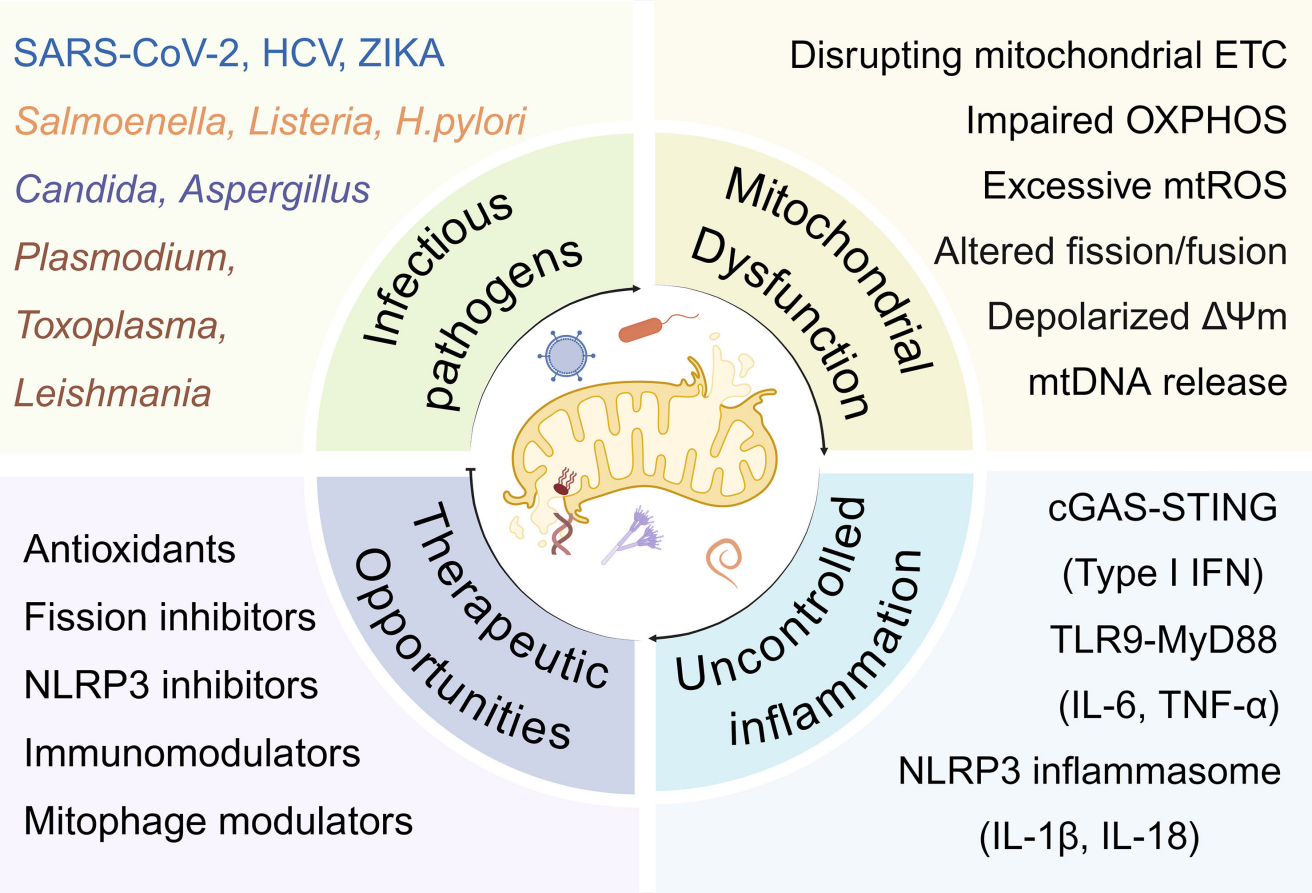
Pathogen-induced mitochondrial dysfunction and therapeutic opportunities. A wide range of infectious pathogens-including viruses (e.g., SARS-CoV-2, HCV, Zika), bacteria (*Salmonella, Listeria, H. pylori*), fungi (*Candida, Aspergillus*), and parasites (*Plasmodium, Toxoplasma, Leishmania*)-can disrupt mitochondrial homeostasis. Pathogen-induced mitochondrial dysfunction involves impairment of the electron transport chain (ETC, reduced oxidative phosphorylation (OXPHOS), excessive mitochondrial ROS (mROS), altered fission and fusion dynamics, loss of mitochondrial membrane potential (ΔΨ), and release of mitochondrial DNA (mDNA). These events activate innate immune pathways such as GAS-STING (TypeIFN response), TLR9-MyD88 (IL-6, TNF-α), and the NLRP3 inflammasome (IL-1β, IL-18), leading to uncontrolled inflammation. Therapeutic opportunities targeting these processes include the use of antioxidants, mitochondrial fission inhibitors, NLRP inflammasome inhibitors, immunomodulators, and mitophagy regulators to restore mitochondrial function and immune balance.

Mitochondria function as central hubs that integrate metabolic regulation with innate immune signaling, coordinating host defense against diverse pathogens. As illustrated in **Figure 1**, viruses (SARS-CoV-2, HCV, Zika), bacteria (*Salmonella, Listeria, H. pylori*), fungi (*Candida, Aspergillus*), and parasites (*Plasmodium, Toxoplasma, Leishmania*) converge on common mitochondrial targets to promote their survival. These pathogens disrupt oxidative phosphorylation, elevate mitochondrial ROS, and alter mitochondrial dynamics, leading to depolarized membrane potential and mtDNA release (Maekawa et al., 2019). The resulting mitochondrial stress activates key innate immune pathways, including cGAS-STING, TLR9, and NLRP3 inflammasome signaling, which collectively determine the balance between protective immunity and immunopathology (Xian et al., 2022). While moderate activation promotes pathogen clearance, dysregulation of these pathways drives chronic inflammation and tissue injury (Xie and Zhu, 2024). Understanding this mitochondrial–immune interface provides the foundation for mitochondria-targeted therapies aimed at restoring immune–metabolic homeostasis during infection.

### 6.6 Therapeutic and translational opportunities targeting mitochondrial dysfunction in infection

Targeting mitochondrial dysfunction offers a promising strategy for HDTs. Mitochondria-targeted antioxidants such as MitoQ, SkQ1, and SS-31 have shown protective effects in preclinical models of viral and bacterial infections (Cervantes-Silva et al., 2021). Beyond antioxidants, pharmacological modulation of mitophagy represents another therapeutic avenue. Activation of the PINK1/Parkin pathway during early infection can promote the clearance of damaged mitochondria and restore cellular homeostasis, whereas excessive activation at later stages may contribute to energy depletion and cell death. Thus, stage-specific regulation of mitophagy may be essential for balancing immune defense and mitochondrial integrity (Huang et al., 2023).

Immune modulation through mitochondrial signaling pathways is gaining increasing attention. Inhibition of the NLRP3 inflammasome using selective small-molecule inhibitors, such as MCC950 or OLT1177, has shown efficacy in suppressing excessive cytokine release and reducing infection-associated inflammation (Coll et al., 2015; Marchetti et al., 2018). Conversely, activation of the cGAS-STING pathway using pharmacological agonists may enhance type I interferon production, thereby strengthening antiviral immunity (Zhang et al., 2024a). The rational combination of these strategies-controlling overactive inflammation while preserving beneficial immune signaling-may allow precise tuning of the host response.

Emerging technologies are further expanding translational possibilities. Nanocarrier-based mitochondrial delivery systems enable targeted administration of antioxidants or signaling modulators directly to infected tissues, improving pharmacokinetics and minimizing off-target toxicity (Han et al., 2019). Moreover, CRISPR-based screening of mitochondrial genes during infection is uncovering novel druggable targets involved in host-pathogen interactions (Holmes et al., 2020; Wei et al., 2021). Collectively, these approaches represent a new era of mitochondria-centered therapeutic innovation, offering the potential to complement traditional antimicrobial therapies and mitigate infection-induced tissue injury.

### 6.7 Future directions and emerging frontiers in mitochondrial infection biology

Despite notable progress in understanding pathogen-induced mitochondrial dysfunction, several critical knowledge gaps remain. One key area is the temporal dynamics of mitochondrial responses, as distinguishing early adaptive changes from late-stage pathological damage requires longitudinal investigations. Another important aspect involves tissue-specific vulnerabilities, since organs such as the lung, liver, and brain may exhibit distinct mitochondrial adaptations to infection. Additionally, host genetic variation, particularly polymorphisms in mitochondrial genes or mitophagy regulators, could significantly influence infection susceptibility, disease progression, and therapeutic outcomes. These dimensions underscore the complexity of host-pathogen interactions at the mitochondrial level and the need for comprehensive, context-specific studies.

Emerging research directions also point to the interplay between mitochondria, the microbiome, and metabolic states, which likely modulates mitochondrial resilience during infection and shapes immune responses. Moreover, the identification of clinical biomarkers, such as circulating mtDNA or mitochondrial proteins, holds promise for improving diagnosis and predicting infection severity. To address these challenges, future studies should leverage multi-omics integration, high-resolution imaging, and systems biology approaches to construct detailed maps of mitochondrial networks under infectious stress. Ultimately, the development of mitochondria-focused interventions could complement existing antimicrobial strategies, mitigating both pathogen virulence and host tissue injury, and offering a new paradigm for precision infection therapy.

## 7 Conclusions

Mitochondria are no longer considered passive energy generators; they function as active participants in host defense and pathogen exploitation. Across viruses, bacteria, fungi, and parasites, targeting mitochondria represents a convergent strategy for immune evasion and survival. Despite diverse mechanisms, key vulnerabilities-including mitochondrial dynamics, ROS homeostasis, and immune signaling-offer opportunities for host-directed therapeutics. Future efforts should aim to develop precision interventions targeting mitochondrial pathways, supported by robust biomarkers and translational models. By bridging mitochondrial biology with infection immunology, this field holds transformative potential for managing infectious diseases and improving clinical outcomes.
